# Disrupted Skeletal Muscle Mitochondrial Dynamics, Mitophagy, and Biogenesis during Cancer Cachexia: A Role for Inflammation

**DOI:** 10.1155/2017/3292087

**Published:** 2017-07-13

**Authors:** Brandon N. VanderVeen, Dennis K. Fix, James A. Carson

**Affiliations:** Integrative Muscle Biology Laboratory, Department of Exercise Science, University of South Carolina, Columbia, SC, USA

## Abstract

Chronic inflammation is a hallmark of cancer cachexia in both patients and preclinical models. Cachexia is prevalent in roughly 80% of cancer patients and accounts for up to 20% of all cancer-related deaths. Proinflammatory cytokines IL-6, TNF-*α*, and TGF-*β* have been widely examined for their regulation of cancer cachexia. An established characteristic of cachectic skeletal muscle is a disrupted capacity for oxidative metabolism, which is thought to contribute to cancer patient fatigue, diminished metabolic function, and muscle mass loss. This review's primary objective is to highlight emerging evidence linking cancer-induced inflammation to the dysfunctional regulation of mitochondrial dynamics, mitophagy, and biogenesis in cachectic muscle. The potential for either muscle inactivity or exercise to alter mitochondrial dysfunction during cancer cachexia will also be discussed.

## 1. Introduction

Pathological inflammation, a hallmark of numerous chronic diseases, can lead to fatal comorbidities, including cachexia [1–4]. Cachexia is characterized by unintentional body weight loss secondary to an underlying disease [1, 3] and is prevalent in ~60–80% of cancer patients. Cancer patients exhibiting cachexia have increased fatigue, decreased functional independence, reduced life quality, and decreased survival [5–10]. Although no treatments are currently approved for cancer cachexia, improving the mechanistic understanding of skeletal muscle mass loss and more recently skeletal muscle metabolic function is thought to be central to the etiology of cancer cachexia and the successful development of therapeutic interventions.

Skeletal muscle mass and metabolism have established roles for maintaining health in obesity, ageing, and chronic disease [11–13]. Related to health, skeletal muscle serves as an amino acid reservoir for the body and a primary site of insulin-stimulated glucose transport [11, 14]. However, skeletal muscle relies heavily on lipids as a fuel source during rest and low-intensity activities and contributes to over 20% of whole body fatty acid metabolism [14]. This oxidative metabolism dependence underscores the muscle mitochondria's critical role in metabolic homeostasis [14, 15]. The analysis of muscle oxidative metabolism involves the quantification of mitochondria content, respiratory capacity, and the efficiency of the Krebs cycle and electron transport chain (ETC) [16, 17]. This line of inquiry has significantly advanced our mechanistic understanding of aging, disease, and physical inactivity's effects on muscle metabolism.

Dysfunctional muscle oxidative metabolism occurs with many disease conditions [12, 13, 18–21] and can involve mitochondrial dynamics, mitophagy, and biogenesis regulation [18, 22]. Each of these dysfunctions is being actively investigated for their role in the pathogenesis of cancer cachexia [12, 13, 23]. Skeletal muscle mitochondrial dysfunction has been reported with cachexia in cancer patients and preclinical models [12, 15, 24–27] and is consistent with functional changes involving increased muscle fatigability and overall weakness [5, 6, 8, 9, 28]. Accelerated catabolism and suppressed anabolism in wasting muscle has been linked to mitochondrial dysfunction [12, 25, 26]. The primary objective of this literature review is to highlight evidence linking cancer-induced inflammation to the regulation of muscle mitochondrial dynamics, mitophagy, and biogenesis. We will stress research areas that warrant further investigation to establish if they are a consequence of cachexia or a cause of the pathology. The examination of inflammatory mediators of cancer cachexia will be delimited to interleukin 6 (IL-6), tumor necrosis factor *α* (TNF-*α*), and transforming growth factor *β* (TGF-*β*) superfamilies' role. Evidence for these cytokines in the overall regulation of cachexia progression and muscle mass loss has been extensively reviewed elsewhere [29–38] and will only be briefly described here. We will also discuss the potential for either muscle inactivity or exercise to alter the regulation of dysfunctional mitochondrial dynamics, mitophagy, and biogenesis during cancer cachexia.

## 2. Overview of Inflammation as a Driver of Cancer Cachexia

### 2.1. Overview

Increased systemic inflammation is an established driver of cachexia development in numerous chronic diseases, including cancer [3]. Several cytokines have been implicated as the mediators of chronic inflammation for cachexia progression in both human and preclinical animal models [1, 29, 39]. Cytokines can regulate intracellular signaling that induces muscle wasting in response to various stimuli. IL-6, TNF-*α*, and TGF-*β* are cytokines that have been mechanistically linked to skeletal muscle wasting and disrupted metabolic homeostasis during cancer cachexia [29–38].

### 2.2. Interleukin-6

**Figure 1:**
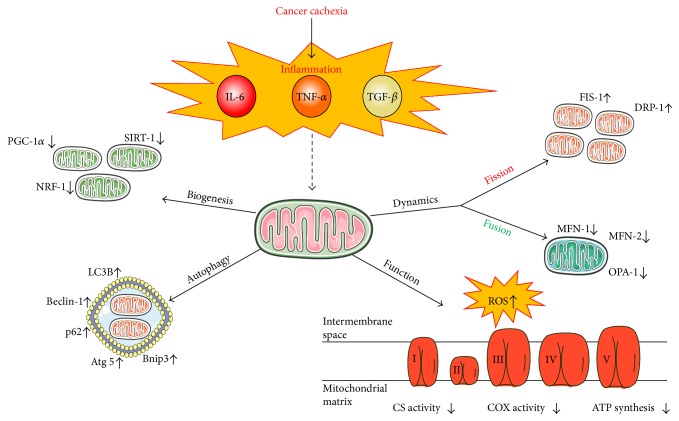
Cancer cachexia-induced inflammation regulates mitochondria. Chronic inflammation during cancer cachexia is associated with increased circulatory proinflammatory cytokines IL-6, TNF-*α*, and TGF-*β*. Chronic inflammation through these cytokines has been demonstrated to decrease mitochondrial biogenesis through decreased activation of PGC-1a, NRF-1, and Sirt-1. Increased autophagy is apparent in cachectic muscle through inducing LC3B, Beclin-1, p62, Atg 5, and Bnip3 and dysregulating dynamics shown by increased FIS-1 and Drp-1 and decreased MFN-1, MFN-2, and OPA-1. These factors contribute to decreased mitochondrial function and ATP synthesis. The figure was made with Servier Medical Art (http://www.servier.com/Powerpoint-image-bank).

The IL-6 cytokine family has been widely investigated in skeletal muscle remodeling due to exercise, aging, and disease [29, 40–43]. IL-6 is a pleiotropic cytokine implicated as a critical regulator of inflammation-induced skeletal muscle and fat wasting during cancer cachexia [35]. Elevated circulating IL-6 can be observed in cachectic cancer patients and preclinical models alike and is strongly correlated to body weight and muscle mass loss [44–46]. IL-6 signals through the ubiquitously expressed glycoprotein 130 (gp130) receptor to activate downstream intracellular signaling pathways [42, 47, 48]. While IL-6 can activate numerous cellular signaling pathways, the phosphorylation of immediate downstream target signal transducer and activator of transcription 3 (STAT3) has been most widely examined with cachexia-induced muscle mass loss [41, 49, 50]. STAT3 activation by IL-6 causes the disruption of skeletal muscle proteostasis through both anabolic and catabolic signaling [51]. STAT3 inhibition can attenuate body weight and muscle mass loss in tumor-bearing mice [52, 53]. This review will discuss the implications for IL-6-induced STAT3 signaling in the regulation of cachexia-induced mitochondrial dysfunction (Figure 1).

### 2.3. Tumor Necrosis Factor *α*

TNF-*α*'s role in muscle wasting during cachexia has been well studied [1, 34, 54–56]. TNF-*α*, released from activated macrophages, can activate skeletal muscle nuclear factor *κ*B (NF-*κ*B) transcription factor and promote protein degradation through the transcription of ubiquitin proteasome E3 ligases, MurF1, and Atrogin1 [34, 38]. Muscle MurF1 and Atrogin1 expression are prevalent in cancer patients and preclinical cachexia models and promote skeletal muscle protein degradation [57]. TNF-*α* can also promote body weight loss through the loss of adipose tissue by stimulating lipolysis and inhibiting lipogenesis [34]. However, TNF-*α* also promotes anorexia [58, 59]. Cancer-induced TNF-*α* levels increase corticotrophin-releasing hormone (CRH), which reduces appetite and food intake [33, 34, 60]. However, TNF-*α* overexpression in mice lacking tumors induced weight loss, which was not different than pair-fed controls [61]. This portion of the review will focus on TNF-*α*'s induction of NF-*κ*B to disrupt mitochondrial homeostasis (Figure 1).

### 2.4. Transforming Growth Factor *β*

TGF-*β* cytokine super family consists of 34 proteins that regulate a myriad of cellular functions. Several family members have been found to promote cancer-induced skeletal muscle wasting [30, 62]. TGF-*β*1, Activin A, TNF like weak inducer of apoptosis (TWEAK), and myostatin are TGF-*β* super family members that bind to either type I or type II activin receptors in skeletal muscle and activate Smad (SMA, mothers against decapentaplegic) signaling [1, 30, 56, 63–66]. Smad regulation of skeletal muscle wasting is still an area of active inquiry, but evidence suggests a role for forkhead box O3- (FOXO3-) dependent protein degradation as well as protein synthesis suppression through protein kinase B (Akt) [30, 38]. Activin A administration can induce the cachectic phenotype in nontumor-bearing mice through Smad2/3 activation, which increases atrophy and fibrotic gene transcription [67]. While TGF-*β* signaling's role in cachexia continues to be elucidated, this review will discuss evidence for the TGF-*β* superfamily to regulate skeletal muscle mitochondria function (Figure 1).

## 3. Mitochondrial Dysfunction in Cachectic Muscle and Inflammatory Mediators

### 3.1. Overview

Cachexia can be defined as a complex metabolic syndrome, and thus skeletal muscle mitochondria have become an intriguing focus for determining the underpinnings of cancer-induced muscle catabolism [3]. To this end, the maintenance of mitochondrial content and capacity for ATP production in cachectic muscle have become active areas of inquiry. While numerous studies have reported mitochondrial content loss with wasting, there remains a need to better define mitochondrial function and the regulators of this process in cachectic muscle. Mitochondrial function is classically defined as the capacity for ATP production through oxidative phosphorylation and beta-oxidation [24, 68, 69]. Disruptions to the ETC decrease mitochondrial respiration and the ability to produce ATP. While relatively few published studies have directly examined muscle mitochondrial respiration with cancer cachexia, cachectic skeletal muscle exhibits decreased cytochrome c oxidase enzymatic activity and oxygen consumption [70, 71].

Inflammatory signaling has been linked to cancer-induced mitochondrial dysfunction in skeletal muscle [12]. Specifically, activation of either NF-*κ*B, STAT3, or Smad3 signaling has been associated with cancer-induced muscle mitochondria dysfunction in tumor-bearing mice. In vivo and in vitro analysis of Lewis lung carcinoma-driven cachexia demonstrated decreased muscle ATP synthesis rates and decreased mitochondrial electron flow with associative increases in TNF-*α* [68, 72]. Furthermore, inhibiting NF-*κ*B signaling improved diaphragm mitochondrial respiration in mice bearing P07 lung-derived tumors [70]. The IL-6 signaling pathway has also been linked to muscle mitochondrial function with cachexia [12]. STAT3 accumulation in isolated liver and heart mitochondria negatively regulates mitochondrial respiration and ATP production through binding to Complex I in the inner mitochondrial membrane and interacting with retinoid-interferon-induced mortality (GRIM) 19 [73]. Reduced enzyme activity in isolated skeletal muscle mitochondria is demonstrated in mice with elevated Smad 3 signaling [71]. Currently, our understanding of muscle mitochondrial respiration during cancer cachexia is extremely limited due to the dearth of published studies and the heterogeneity of the preclinical cancer cachexia models used in these investigations. However, further mechanistic inquiries into both the drivers of mitochondrial dysfunction and the ramifications of this dysfunction for muscle wasting and functional decline are warranted.

Mitochondrial dysfunction has been tightly associated with excess production of reactive oxygen species (ROS) [72]. While ROS generation is involved in muscle cellular signaling that supports cell homeostasis [72], chronically elevated ROS can initiate DNA damage, protein oxidation, and apoptosis [70, 74, 75]. To this end, substantial evidence points to increased ROS production in cachectic skeletal muscle [76–78]. The role for ROS to promote skeletal muscle dysfunction and atrophy is well established and has been reviewed extensively [79–85]. Although elevated ROS has been identified in wasting skeletal muscle, it has not yet been determined if ROS initiates muscle catabolism in cancer cachexia or is a consequence of the wasting process [82].

### 3.2. Mitochondrial Dynamics

Understanding skeletal muscle mitochondrial dynamics during cancer cachexia has become an extremely active area of investigation. While initial studies focused on describing changes to mitochondrial dynamics in cachectic muscle, recent research has begun to elucidate the drivers of disrupted mitochondrial dynamics in cachectic muscle and the ramifications this disruption has on muscle mass loss and metabolic dysfunction [12, 13, 25]. The interconnected muscle mitochondrial network undergoes tightly regulated processes related to fusion and fission, which are coordinated to influence mitochondrial homeostasis [13, 86, 87]. The fusion of mitochondria induces extension of the mitochondrial network thought to increase energy efficiency and increase ATP production [20]. Conversely, the process of fission involves the fragmentation of mitochondria and segregates damaged areas of the mitochondrial network that may be dysfunctional, allowing for their removal [86–90]. Mitochondrial dysfunction can result from the disrupted coordination of fission and fusion processes; several preclinical models of cancer cachexia and cancer patients have demonstrated altered indices of mitochondrial fission and fusion [91–93].

Mitochondrial dynamics' processes have been extensively studied and characterized both in vivo and in vitro and have been previously reviewed [13, 90, 94, 95]. The fusion process is regulated by mitofusin 1 and 2 (MFN-1, MFN-2) and optic atrophy protein 1 (OPA1) [88, 95]. While these proteins are similar in structure, their functions are thought to be nonredundant. MFN-1 regulates GTP tethering whereas MFN-2 regulates the assembly of the fusion complexes [12, 13]. OPA-1 is expressed as several different isoforms and is necessary for the regulation of fusion GTP tethering in conjunction with MFN-1 [87]. The loss of mitochondrial fusion has detrimental effects in skeletal muscle shown by genetic knockout of MFN-1 and 2 resulting in muscle atrophy and reduced mitochondrial DNA (mtDNA) [96].

Circulating IL-6 and muscle STAT3 signaling have been linked to suppressed MFN-1 expression in cachectic muscle. Systemic IL-6 overexpression in *Apc^Min/+^* suppressed MFN-1 expression, but was rescued by administration of an IL-6 receptor antibody [92]. Additionally, IL-6 administration to cultured myotubes increased STAT3 activation and suppressed MFN-2 in a dose-dependent manner [92]. Similarly, TNF-*α* was able to decrease myotube MFN-2 expression associated with elevated ROS and reduced ATP production [97].

**Figure 2:**
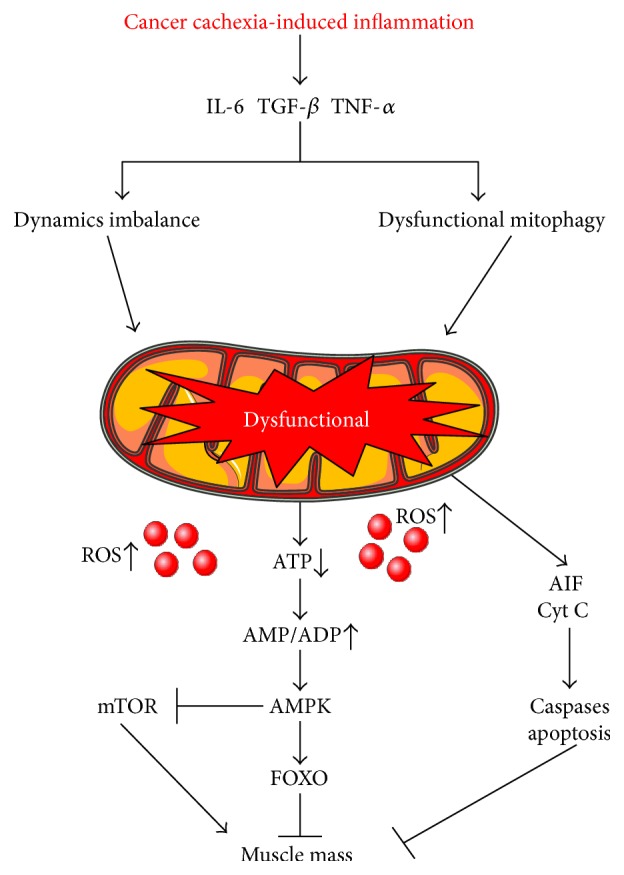
Mitochondrial dysfunction in skeletal muscle negatively regulates muscle mass. Elevated IL-6, TNF-*α*, and TGF-*β* during cancer cachexia disrupt mitochondrial homeostasis leading to dysfunction mitochondria. Dysfunctional mitochondria release aberrant amounts of reactive oxygen species (ROS) and decrease ATP production. This leads to chronic activation of AMPK to negatively regulate protein synthesis causing decreased muscle mass. The figure was made with Servier Medical Art (http://www.servier.com/Powerpoint-image-bank).

Mitochondrial fission is necessary for skeletal muscle mitochondria maintenance and quality [13, 86, 89, 90, 98]. Mitochondrial fission machinery is controlled by the GTPase cytosolic dynamin-related protein 1 (DRP-1) which can translocate to the outer mitochondrial membrane and develop active fission sites [13, 86, 87]. DRP-1 can be regulated by phosphorylation and sumoylation by small ubiquitin-related modifiers (SUMOs) [13]. Fission protein 1 (FIS-1) is proposed to be required for mitochondrial division as it serves to recruit DRP-1 to the outer mitochondrial membrane [87]. Accelerated fission results in proapoptotic signals that lead to mitochondria isolation from the network and reduces its ATP efficiency [90]. Interestingly, accelerated mitochondrial fission is associated with AMPK activation, which can stimulate mitochondrial biogenesis in healthy muscle [86, 99]. However, while accelerated fission is often regarded as a sign of mitochondrial dysfunction in inflammatory diseases [12, 77, 89, 92], failure to undergo fission will result in mitochondrial dysfunction and muscle atrophy [86, 89, 92] (Figure 2()).

Although evidence suggests an important role, the direct effects of inflammation on mitochondrial fission continue to be established. Systemic IL-6 overexpression in *Apc^Min/+^* mice had elevated FIS-1 protein levels prior to the onset of cachexia [92]. Interestingly, IL-6-induced muscle FIS-1 expression is not selective to muscle phenotype as it occurs equally in both highly oxidative and highly glycolytic fibers [77, 92]. While a direct link between TNF-*α* and skeletal muscle mitochondrial fission is not well established, the TNF-*α* induction of ROS provides intriguing rationale. Interestingly, overexpression of FIS-1 in healthy animals has been demonstrated to be proapoptotic and is tightly associated with accelerated production of ROS [86]. However, it is not well understood if elevated ROS production is causal or consequence of disrupted mitochondrial dynamics in cachectic muscle [100].

### 3.3. Mitophagy

Hyperactivation of cellular degradation pathways has become an established target of chronic inflammatory conditions [101, 102]. Autophagy in cachectic muscle has become widely investigated for the regulation of skeletal muscle mass loss and disrupted metabolism [38, 103, 104]. Autophagy is a highly conserved cellular process that contributes to the lysosomal degradation of proteins and organelles (including mitochondria) that are either dysfunctional, damaged, long lived, or misfolded [88, 105]. The process of autophagy consists of a small portion of the cytoplasm that includes organelles or proteins being sequestered by a phagophore to form an autophagosome. This autophagosome will then fuse with the lysosome to become an autolysosome which then degrades the cellular cargo contained within it. These processes require a family of proteins known as autophagy-related genes (Atgs) which are important in the signaling and regulation of autophagy [106–111]. Autophagy can be both a nonselective process (e.g., starvation) and a highly selective process that degrades specific organelles such as mitochondria which has been termed mitophagy [12, 106, 107]. The selectivity of this process can be determined by specific proteins, p62 and BCL2 interacting protein 3 (Bnip3), which have cargo-binding domains and LC3-interacting domains which are responsible for recruitment and binding of autophagosome proteins [13, 90, 112]. The autophagic removal of damaged and dysfunctional mitochondria, mitophagy, is critical for maintaining a healthy network of mitochondria. Failure of these processes can lead to an accumulation of damaged mitochondria which can negatively regulate metabolism and mass [13, 110, 113].

Increased lysosomal protease activity, indicative of accelerated autophagy flux, has been reported in cachectic muscle from tumor-bearing mice [113, 114]. Interestingly, circulating branched chain amino acids are elevated in cancer patients prior to weight loss, suggesting accelerated autophagy is an early event in cachexia development [115]. Tumor growth is associated with a reduced nutrient availability, and it has been suggested that tumor-derived factors can accelerate mitophagy [116]. Accelerated mitophagy has the potential to contribute to skeletal muscle mitochondrial dysfunction [92, 117, 118]. Skeletal muscle from cancer patients and preclinical models of cancer cachexia (*Apc^Min/+^*, C26, and LLC) have demonstrated accelerated mitophagy indices [92, 101, 104, 119–121]. Muscle mitophagy can occur through an AMPK, FOXO, and mTORC1 signaling axis, which are established regulators of both muscle metabolism and mass; the cachectic environment also disrupts this signaling axis [110, 121, 122] (Figure 2). Tumor necrosis receptor factor 6 (TRAF6) is a potent inducer of mitophagy, and TRAF6 deletion can prevent cancer-induced muscle mass in tumor-bearing mice [123]. Interestingly, both Activin A and TWEAK have identified roles in the modulation of LC3, potentially indicating disrupted mitophagy [56, 67]. Recently, tumor-derived factors released into circulation were shown to induce mitophagy in skeletal muscle through IL-6-dependent signaling [124]. The autophagy inducing bioactivity of serum collected from gastrointestinal and lung cancer patients was significantly correlated to weight loss, but was normalized with the administration of an IL-6 receptor antibody [124]. Together, current evidence suggests that during cancer cachexia, tumor-secreted IL-6 has an important role in mitophagy regulation. Further, work is warranted to determine if disrupted mitophagy regulation is a viable therapeutic target for cancer-induced muscle wasting or if mitophagy is being induced in cachectic muscle to correct other metabolic dysfunctions.

### 3.4. Mitochondrial Biogenesis

Muscle adaptation to increased and decreased use provides a clear demonstration that healthy skeletal muscle fiber's mitochondria content is plastic and reflects the fiber's energy requirements [13, 125–127]. However, chronic inflammation can create an environment that disrupts this regulation to incite the loss of muscle oxidative metabolic capacity [128–131]. Mitochondrial biogenesis is a critical process for maintaining the necessary mitochondria content to meet energy demands [13, 125, 127, 132]. The peroxisome-proliferator gamma-activated receptor (PGC-1) has been extensively examined as a critical regulator of muscle mitochondrial biogenesis. There are several PGC-1 isoforms, and each has significant but independent roles in oxidative metabolism. PGC-1*α*4 regulates muscle protein synthesis through IGF-1 and myostatin signaling cascades [133, 134]. PGC-1*β* can regulate myosin heavy-chain isoform expression, and increased expression induces an oxidative muscle phenotype [135]. PGC-1*α* can induce nuclear response factors (NRF-1, NRF-2) and mitochondrial transcription factor A (Tfam) transcription, which regulate mitochondrial biogenesis [125–127, 136]. Moreover, PGC-1*α* loss results in reduced muscle mitochondrial content and ATP production [137–139].

Increased PGC-1*α* expression is protective against muscle atrophy in aging, decreased use, and inflammatory cytokine administration. However, the limited number of investigations in preclinical cancer cachexia models is equivocal [140–143]. While suppressed PGC-1*α* expression is consistently reported in cachectic skeletal muscle [77, 92, 144], overexpression was not sufficient to prevent Lewis lung carcinoma- (LLC-) induced muscle wasting [145]. Interestingly, PGC-1*α* overexpression could stimulate mitochondrial biogenesis in the cachectic muscle, indicating that the pathways to induce mitochondrial biogenesis were functional in the cachectic environment. Regulators of PGC-1*α* activity are also major determinants of muscle metabolic capacity in both healthy and cachectic muscle [146]. AMPK, an energy stress sensor, regulates muscle oxidative metabolism through PGC-1*α*-regulated biogenesis and ULK-dependent mitophagy [147–149]. In healthy muscle, activated AMPK can stimulate mitochondrial biogenesis and has been demonstrated as therapeutic in type II diabetes [149, 150]. Muscle AMPK is chronically activated in some preclinical cancer cachexia models, however, fails to induce mitochondrial biogenesis. Interestingly, chronic AMPK activation in cachectic muscle may have a role in the suppression of muscle protein synthesis [151]. Circulating IL-6 has been associated with muscle AMPK activation in the cachectic *Apc^Min/+^* mouse. IL-6 overexpression in tumor-bearing mice can activate AMPK and reduce PGC-1*α* expression, whereas IL-6 receptor antibody administration attenuates cancer-induced AMPK activation [151]. Although in vivo evidence for the direct effects of IL-6 signaling on cachectic muscle AMPK activation is lacking, IL-6 administration to skeletal muscle myotubes can directly activate AMPK [151]. Further research is needed to understand the disrupted feedback caused by the cachectic environment that uncouples AMPK signaling from mitochondrial biogenesis.

Activin A and myostatin have the potential to also disrupt mitochondrial biogenesis. Ge et al. demonstrated that the lack of Smad3 signaling resulted in decreased NRF and Tfam activation [152]. However, Smad3 activation via the TGF-*β* super family in cachectic skeletal muscle remains to be determined. Interestingly, both Activin A and TWEAK were able to disrupt mitochondrial biogenesis by reducing PGC1-*α* [67, 153]. Further research is needed to establish if this mitochondrial biogenesis suppression is a therapeutic target for either preventing muscle mass loss or improving metabolic health during cancer cachexia.

## 4. Exercise Countermeasures to Cancer-Induced Mitochondrial Dysfunction

The capacity to regenerate from injury and adapt to altered use are defining features of skeletal muscle that also provide optimism for therapeutic interventions for cachectic muscle. Exercise has shown to be beneficial in diabetes, COPD, and CHF and continues to show beneficial results in cancer patients as well [154, 155]. Activity level can dramatically impact skeletal muscle mass and metabolism [88, 156]. Increased muscle activity can also induce a more oxidative muscle phenotype by increasing mitochondria content and function [95, 157–159]. Increased muscle use can positively impact muscle mass, and the extent of this change is dependent on the exercise type, intensity, duration, and frequency [5, 160]. The metabolic plasticity of muscle is reinforced by the dramatic alterations that occur to skeletal muscle after an acute bout of exercise [88, 156, 157]. Increasing the muscle metabolic demand with exercise can stimulate mitochondrial biogenesis to increase mitochondrial content and function [138, 139, 161]. Cachectic muscle from tumor-bearing mice subjected to an acute bout of low frequency electrical stimulation maintains the capacity to activate genes responsible for mitochondrial biogenesis, PGC-1*α*, NRF-1, and Tfam [144]. However, cachectic muscle had deficits in the acute activation of protein expression after a single bout of stimulated concentric contractions, which could be rescued by systemic inhibition of inflammatory signaling [144].

**Figure 3:**
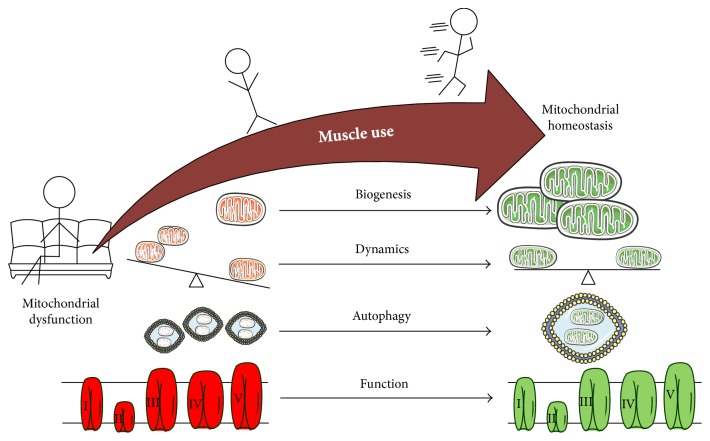
Increased muscle use improves skeletal muscle mitochondrial homeostasis in healthy and cachectic conditions. Sedentary behavior or muscle disuse is associated with decreased mitochondrial biogenesis, improper balance of mitochondrial dynamics, dysregulation of autophagy, and mitochondrial dysfunction. Increasing muscle use by reducing sedentary behavior or exercise will increase mitochondrial biogenesis, regulate mitochondrial dynamics, improve autophagic flux, and improve mitochondrial function and ATP efficiency. Overall increased muscle use will lead to skeletal muscle mitochondrial and metabolic homeostasis. The figure was made with Servier Medical Art (http://www.servier.com/Powerpoint-image-bank).

Decreased muscle use, either by unloading or extreme sedentary behavior, can induce a shift to a more glycolytic phenotype, coinciding with decreased mitochondrial content and function and muscle atrophy [155]. Cancer patients commonly suffer from excessive fatigue prior to and during treatments [5, 6, 162]. This fatigue is accompanied by a dramatic decrease in physical activity and the ability to perform activities of daily living (ADLs) [5, 163–165]. Preclinical cancer models also have shown that cachectic mice undergo limited volitional activity [166, 167]. However, minimizing sedentary time and using alternative muscle contraction methods may serve as a first line of action to attenuate cachexia-induced decrements in muscle mitochondria [168–171] (Figure 3). IL-6 overexpression in tumor-bearing mice was not able to induce muscle mass loss and metabolic changes when they were regularly exercised on a treadmill [172]. It is interesting to speculate if disuse alters the muscle sensitivity to the cachectic environment, causing a more rapid decline in muscle metabolic function and mass. Conversely, research is needed to determine if muscle contraction or exercise serves to desensitize the muscle to the cachectic environment.

Exercise also regulates mitochondrial dynamics, increasing both fission and fusion. This is thought to aid in mitochondrial turnover and improve efficiency (Figure 3). Similarly, autophagy flux increases after an acute bout of exercise. IL-6 overexpression in exercising tumor-bearing mice was resistant to muscle mass loss and metabolic changes [172]. Cachectic muscle in tumor-bearing mice also retains the capacity to respond to repeated bouts of stimulated eccentric contractions. Cachectic muscle in *Apc^Min/+^* mice undergoing 7 bouts of eccentric contractions increased muscle succinate dehydrogenase activity and decreased AMPK signaling [173]. Exercise training is implicated as a potential therapeutic to either prevent or reverse muscle wasting. It is evident from preclinical studies that cachectic muscle maintains the ability to robustly respond to an acute bout of exercise or contraction. Further work is needed to determine if repeated bouts of exercise can confer the metabolic health benefits of exercise after the development of cancer cachexia [139, 174].

Physical activity and contraction has been established as a potent regulator of mitophagy and may possess the potential to correct or attenuate dysfunction mitophagy processes in cachectic muscle [88, 105, 175–179]. In C26 tumor-implanted mice, voluntary wheel running attenuated cachexia-induced p62 and LC3 II/I accumulation indicating improved mitophagy [179]. Additionally, AMPK activation via AICAR suppressed p62 accumulation through promotion of mitophagy and accelerating the turnover of p62 accumulation in cachectic muscle [179]. While there is growing evidence for mitophagic processes in the regulation of cancer cachexia, additional studies are warranted to establish a direct role for inflammation in the regulation of these processes and to clearly examine mitophagy flux in vivo. Additionally, the role of exercise and/or muscle contraction in the regulation of mitophagy in diseased or chronically inflamed muscle may prove to be a powerful therapeutic for the restoration of mitophagic balance in cachectic muscle. Clearly, further research is warranted to examine the complex interaction between cancer-induced inflammation, muscle contraction, and muscle disuse for maintenance or improvement of cachectic muscle oxidative metabolism.

## 5. Conclusions

Disrupted mitochondrial homeostasis contributes to the loss of functional capacity in cancer patients and can negatively impact the quality of life and survival. Skeletal muscle mitochondria not only regulate oxidative metabolism but also proteostasis and muscle mass maintenance. The role of mitochondria in skeletal muscle wasting during cancer cachexia has emerged as novel investigative target in cachexia studies. There is a clear relationship between inflammation and mitochondrial dysfunction related to dynamics, mitophagy, and biogenesis. While the alterations to mitochondrial dynamics during cachexia appear evident, a more mechanistic approach is necessary to understand regulatory mechanisms and functional outcomes. A growing body of research suggests an important therapeutic strategy involving the reduction of muscle disuse and increasing muscle contractile activity for the maintenance of skeletal muscle metabolic health, even in the presence of a cachectic environment. To this end, analysis of functional and metabolic outcomes, muscle strength, and fatigability are necessary to understand the totality of the cachectic condition.
